# Infectious and inactivated foot-and-mouth disease virus serotypes A/ARG/2001 and O1 Campos/Bra/58 differentially affect bovine afferent lymph dendritic cells

**DOI:** 10.3389/fimmu.2026.1909847

**Published:** 2026-10-27

**Authors:** Valeria Quattrocchi, Ivana Soria, Cecilia Ana Langellotti, Mariela Gammella, Juan Esteban Bidart, Sergio Ferraris, Jorge Ernesto Carrillo, Fernando Lange, Verónica Maldonado, Lucas Vagnoni, Bryan Charleston, Patricia I. Zamorano

**Affiliations:** 1 Instituto de Virología e Innovaciones Tecnológicas (IVIT), Instituto Nacional de Tecnología Agropecuaria (INTA) Castelar, Hurlingham, Buenos Aires, Argentina; 2 Consejo Nacional de Investigaciones Científicas y Técnicas (CONICET), Cdad. Autónoma de Buenos Aires, Argentina; 3 Centro de Ciencias Veterinarias, Universidad Maimónides, Cdad. Autónoma de Buenos Aires, Buenos Aires, Argentina; 4 Centro de Investigaciones en Agroindustrias (CIA) – INTA Castelar, Hurlingham, Buenos Aires, Argentina; 5 INTA Agencia de Extensión Rural (AER) Maipú Estación Experimental Agropecuaria (EEA) - Cuenca del Salado, Maipú, Buenos Aires, Argentina; 6 The Pirbright Institute, Woking, United Kingdom

**Keywords:** A/ARG/2001, ALDCs, differential impact, FMDV, O1 Campos/Bra/58

## Abstract

**Introduction:**

Dendritic cells (DCs) are central to the initiation of adaptive immune responses by bridging innate and adaptive immunity through antigen presentation to naïve T cells. Current vaccines against Foot-and-Mouth Disease (FMD) rely on inactivated virus, although infectious and inactivated forms of FMDV can differentially modulate immune function. While these effects have been studied in murine DCs, their impact on bovine dendritic cells remains poorly understood. This study evaluated how antigen form and serotype affect bovine afferent lymph dendritic cells (ALDCs).

**Methods:**

ALDCs collected from bovine lymph, a physiologically relevant migrating DC population, were exposed to infectious or inactivated FMDV serotypes A/ARG/2001 (A2001) and O1 Campos/Bra/58 (O1C), both included in the commercial vaccine used in Argentina. The expression of MHC class II and the co-stimulatory molecules CD40 and CD86, apoptosis, IL-12 production, and lymphocyte proliferation in co-culture assays were evaluated. Viral progeny production and expression of the non-structural protein 3ABC were also assessed.

**Results:**

Infectious A2001 downregulated MHC class II, CD40, and CD86 expression and increased apoptosis, whereas infectious O1C selectively downregulated MHC class II without affecting ALDC viability. In contrast, inactivated viruses from both serotypes upregulated CD40 and CD86 expression, induced IL-12 production, and promoted lymphocyte proliferation in co-culture assays. No infectious progeny was detected in lymph cells following exposure to infectious virus, although expression of the non-structural protein 3ABC was observed in ALDCs exposed to infectious A2001.

**Discussion:**

These findings provide insight into how antigen form and serotype modulate dendritic cell function in cattle, contributing to a better understanding of the immunological basis for FMDV vaccination strategies in livestock.

## Introduction

Foot and Mouth Disease (FMD) is an acute viral disease caused by the Foot and Mouth Disease virus (FMDV). This virus belongs to the Aphthovirus genus within the Picornaviridae family (1). It affects cloven-hoofed animals including cattle, sheep, goats, pigs and numerous wild species (2). FMD has occurred worldwide, with the exceptions of Greenland, Iceland, New Zealand and the islands of Oceania. It is endemic in various parts of Asia, the Middle East and Africa (3).

FMDV is classified into seven antigenically distinct serotypes: O, A, C, SAT1, SAT2, SAT3, and Asia1, each exhibiting different pathological effects and epidemiological characteristics (4). In South America, serotypes O and A are of greatest concern. These serotypes have caused multiple outbreaks across countries including Colombia, Paraguay, Argentina, Brazil, Uruguay and Venezuela over the past two decades. (5–7).

The virus enters the host primarily via inhalation, initiating replication in the epithelium of the soft palate and pharynx, followed by systemic spread and secondary replication in epithelial tissues of the feet and mouth, which leads to the characteristic vesicular lesions (8). Although mortality in adult cattle is generally low (5% up to 50% in young cattle if the heart muscle is affected), the disease causes significant economic losses due to high morbidity, decreased productivity and the costs of control measures. For countries that export animals and their products, such as Argentina, the major economic losses are due to trade embargoes and restrictions on international commerce.

Vaccination with oil-adjuvanted inactivated FMDV remains the cornerstone for disease control (9). However, the immune response elicited by infection differs from that induced by vaccination: natural infection triggers a rapid T-independent response (10), whereas vaccination typically induces a slower, T cell dependent response (11, 12). Moreover, immunity is largely serotype specific, limiting cross-protection.

Despite numerous studies, the mechanisms involved in the pathogenic process are not completely elucidated, and the ways in which the virus regulates the immune system should be extensively explored.

Given the central role of antigen-presenting cells in shaping the antiviral immunity, DCs represent a key target for understanding how FMDV infection and vaccination drive divergent immune outcomes. Dendritic cells (DCs) are essential antigen-presenting cells that initiate adaptive immunity by capturing, processing, and presenting antigens to naïve T cells. Upon antigen encounter, immature DCs upregulate co-stimulatory molecules (CD80/CD86, CD40 and MHC class II among others), lose endocytic capacity, and migrate to lymph nodes to activate T cells (13, 14). Our group has shown in a murine model that infectious and inactivated FMDV have distinct effects on DC function, which influence the resulting immune response (15, 16). Similar findings have been reported in natural hosts (12, 17–20). However, direct comparisons of infectious and inactivated FMDV on bovine DCs remain unexplored.

Most studies on bovine dendritic cells (DCs) rely on monocyte-derived DCs (MoDCs) generated *in vitro* due to their accessibility. However, MoDCs may not fully reflect the functional properties of naturally circulating DCs, as they arise under inflammatory conditions and display limited migratory capacity to lymph nodes (8, 21–23). Moreover, *in vitro* differentiation conditions can influence DC maturation and function (24). An alternative approach is the isolation of afferent lymph dendritic cells (ALDCs) via cannulation of pseudo-afferent lymphatic vessels, draining bovine skin (21). ALDCs represent DCs that are actively migrating *in vivo* under physiological conditions, providing a more relevant model to study antigen-presenting cell responses in the natural host. ALDCs isolated from lymph have been well characterized and utilized in previous immunological studies (25–27).

Understanding how infectious and inactivated FMDV interact with natural host DCs is crucial for improving vaccine design. In addition, exploring whether different viral serotypes differentially modulate these cells is of particular interest, especially in the case of O1C and A2001, which are epidemiologically relevant in Argentina. In this context, the present study evaluates how infectious and inactivated FMDV serotypes A2001 and O1C modulate bovine ALDCs, providing insight into antigen-presenting cell responses that contribute to the initiation of adaptive immunity.

## Materials and methods

### Collection and characterization of ALDCs

Two Holando-Argentino calves (2–3 month-old with body weights ranging from 90 to 120 kg at the time of lymph node extraction) were utilized to collect afferent lymph cells containing ALDCs. For lymph collection we follow the procedure described in previous studies (21). Briefly: prescapular lymph nodes were surgically removed two months prior to the cannulation procedure. During the cannulation surgery, a cannula was inserted into the vessel formed by the anastomosis of afferent and efferent vessels. Lymph was collected in a flask containing heparin and antibiotics, with the flasks being replaced daily. Lymph cells were concentrated by centrifugation on a histopaque-1083 (Sigma) gradient. Subsequently, the cells were cryopreserved with 10% DMSO in fetal calf serum under liquid nitrogen until further use. Afferent lymph collected contained a mixed population of leukocytes, including T and B lymphocytes, monocytes, NK cells, granulocytes, and a substantial proportion of dendritic cells (10–15%), as previously described. ALDCs were characterized by flow cytometry as DEC205^+^/FSC^high^/CD11c^+^/CD8^-^ (21, 24) Both animals were vaccinated as part of the official national FMD vaccination campaign conducted under SENASA regulations, receiving a single 2 ml dose of the inactivated, oil-adjuvanted Bioaftogen^®^ vaccine (Biogénesis-Bagó), containing FMDV serotypes O1 Campos, A24 Cruzeiro, A Argentina 2001, and C3 Indaial, approximately 4-5 months prior to cannulation (**Figure 1A**). Calves were maintained under controlled conditions during lymph collection, with no opportunity for natural exposure to FMDV. Four calves of the same breed and comparable age and weight followed the same experimental procedure and were successfully cannulated; however, only two calves yielded sufficient lymph for the experiments described in this study.

**Figure 1:**
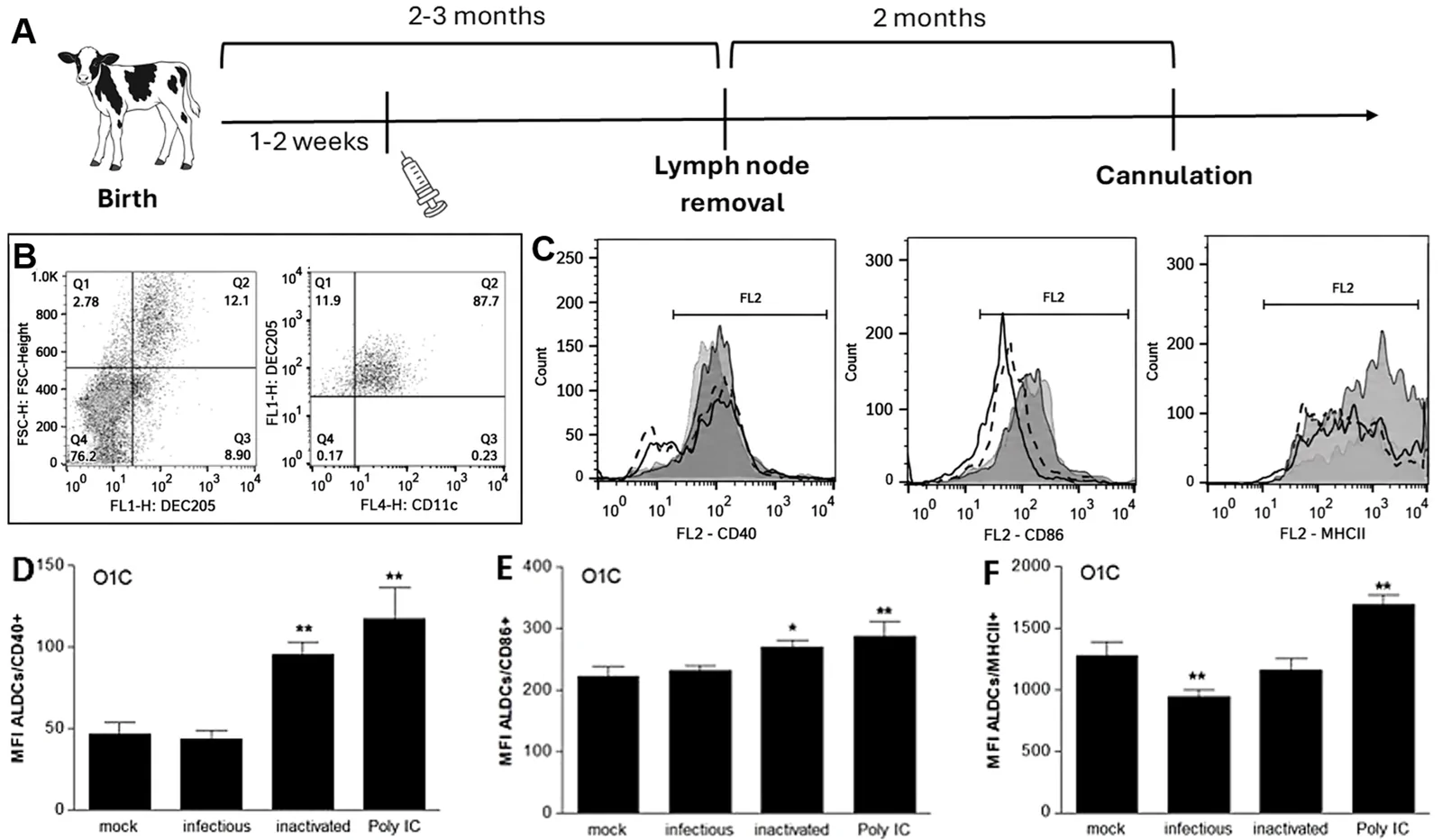
Experimental timeline for calf vaccination, prescapular lymph node removal, and cannulation and *in vitro* expression of co-stimulatory and MHCII molecules in ALDCs stimulated with FMDV serotype O1 Campos. **(A)** Calves received a 2 ml dose of a commercial inactivated, oil-adjuvanted FMD vaccine containing four FMDV strains a few weeks after birth. Prescapular lymph nodes were then surgically removed (2–3 months after birth), and cannulation surgery was performed 2 months later. **(B)** Gating strategy. FSChigh/DEC205 cells were selected in the Q2 quadrant, followed by selection of DEC205/CD11c cells for analysis of co-stimulatory molecule expression. **(C)** Representative histograms showing surface expression of CD40, CD86, and MHCII (left to right). Solid line: mock; dotted line: infectious virus; light gray: inactivated virus; dark gray: Poly I:C (positive control). **(D–F)** Bar graphs represent the mean fluorescence intensity (MFI) ± SEM for ALDCs incubated with: culture medium (mock), inactivated FMDV (inactivated), infectious FMDV (infectious), or Poly I:C. D) CD40 **(E)** CD86 and **(F)** MHCII expression. *p < 0.05. **p < 0.01.

### Isolation of peripheral blood mononuclear cells

Heparinized blood was collected from each cannulated animal through jugular vein puncture. The blood sample was subjected to centrifugation on a Lymphoprep (Ficoll-Paque Plus 1.077 g/ml, GE Healthcare) gradient. The resulting white band, which contained PBMCs, was carefully collected, washed, and cryopreserved with 10% DMSO in fetal calf serum under liquid nitrogen until further use.

### Inactivated and infectious virus production

To produce the inactivated virus, FMDV serotype A/ARG/2001 (A2001) or O1 Campos/Bra/58 (O1C) were cultured in BHK-21 cell cultures within the high biosecurity facilities at Biogénesis-Bagó. The inactivation process was performed using Binary Ethylenimine (BEI) treatment, and the resulting inactivated FMDV (iFMDV) was purified through ultrafiltration/diafiltration. On the other hand, infectious virus was obtained from vesicles of infected animals and propagated in primary cultures of bovine thyroid cells to generate a higher volume of high-titer viruses. To maintain the natural utilization of cell surface αv integrins instead of heparan sulfate proteoglycan, BHK21 passages were avoided. To assess the receptor usage of the infectious FMDV preparation, the virus was inoculated onto monolayers of BHK-21 and CHO cells. BHK-21 cells express both heparan sulfate and integrin receptors used by FMDV for cell entry, whereas the CHO cells used in this assay lack the relevant integrin receptors. Productive viral replication was detected in BHK-21 cells but not in CHO cells. These results were consistent with the ability of the virus preparation to use integrins for cell entry, rather than relying exclusively on heparan sulfate.

Both inactivated and infectious viruses were used in at equivalent amounts, corresponding to a multiplicity of infection (MOI) of 5. For the inactivated virus, the amount used was calculated based on their viral titer prior to inactivation. Mock groups were incubated with culture medium obtained from cells that had not been exposed to infectious virus.

### Assessment of co-stimulatory molecule expression on ALDCs

Lymph cells were incubated for 16 hs with infectious or inactivated FMDV serotype A2001 or O1C. A mock group, consisting of lymph cells incubated with supernatant collected from non-infected BHK-21 cells, was included as a negative control, and Poly I:C as a positive control. The incubation was carried out in IMDM (Gibco) cell culture medium supplemented with 10% fetal calf serum (FCS). The regulation of co-stimulatory molecules CD40, CD86, and MHCII was assessed by performing an indirect surface staining. Mouse monoclonal antibodies against CD40, CD86, and MHCII (SEROTEC) were used, followed by the addition of anti- mouse IgG-Pe conjugated antibodies (BD). Additionally, anti-DEC205 FITC-conjugated and anti-CD11c APC-conjugated monoclonal antibodies were included. The cells were then fixed with 0.2% paraformaldehyde and analyzed using a FACScalibur cytometer and CellQuest software (BD). The expression of co-stimulatory molecules was specifically detected in the FSC^high^/DEC205+/CD11c+ cell population. The gating strategy is shown in **Figures 1**, **2**.

**Figure 2:**
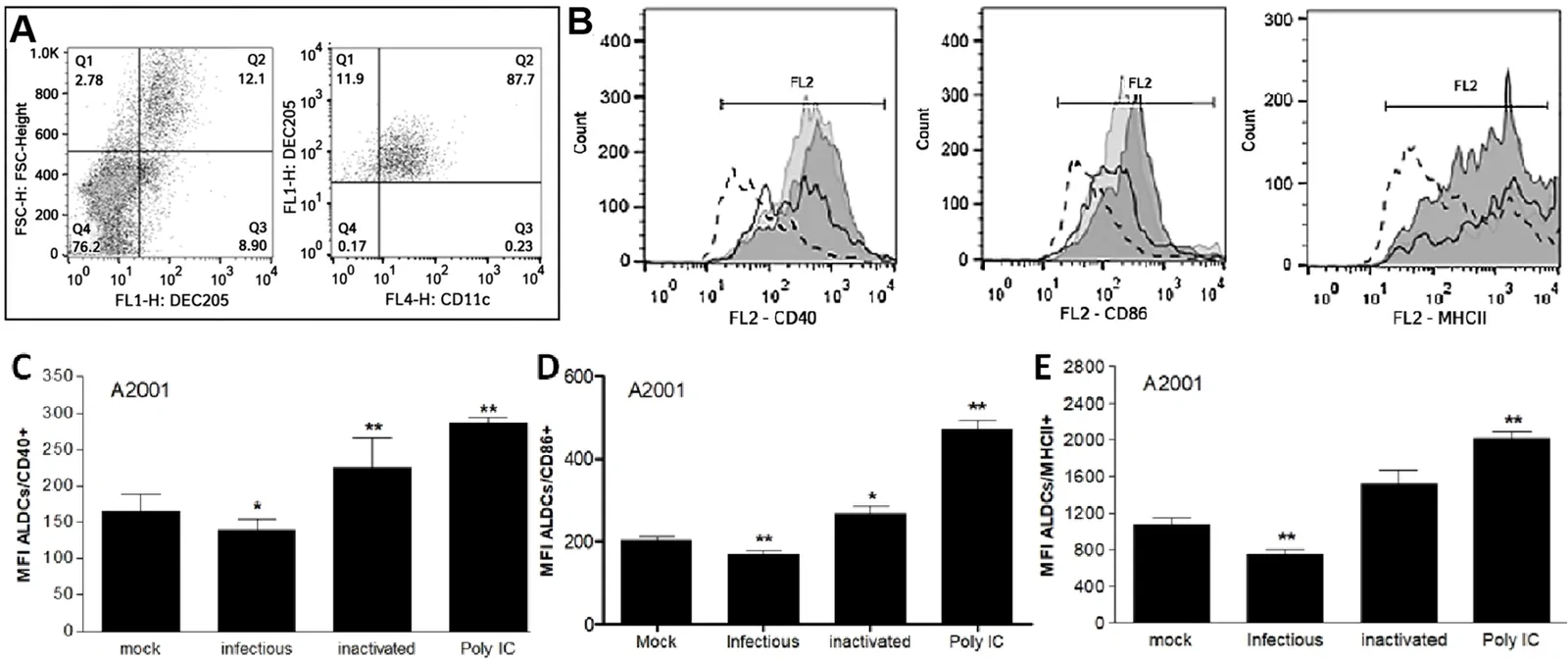
Expression of co-stimulatory and MHCII molecules in ALDCs stimulated with FMDV serotype A2001. **(A)** Gating strategy as shown in **Figure 1A**. **(B)** Representative histograms showing surface expression of CD40, CD86, and MHCII (left to right). Solid line: mock; dotted line: infectious virus; light gray: inactivated virus; dark gray: Poly I:C (positive control). **(C–E)** Bar graphs represent the mean fluorescence intensity (MFI) ± SEM for ALDCs incubated with: culture medium (mock), inactivated FMDV (inactivated), infectious FMDV (infectious), or Poly I:C. **(C)** CD40 **(D)** CD86 and **(E)** MHCII expression. *p < 0.05. **p < 0.01.

### Analysis of lymphocyte proliferation

Inactivated or infectious FMDV from both serotypes, A2001 or O1C, were incubated with lymph cells overnight. After incubation, the cells were washed three times with acidic PBS (pH = 5.5) to remove any virus that was not internalized by the ALDCs. Subsequently, the cells were washed with fresh RPMI 1640 medium (Gibco) supplemented with 10% FCS. The lymph cells, which were incubated with the virus, were then mixed with carboxyfluorescein succinimidyl ester (CFSE) labeled autologous PBMCs (obtained from the same animal). The proliferative response of PBMCs was evaluated by monitoring the progressive reduction in CFSE staining using flow cytometry after 5 days of incubation at 37 °C in a CO_2_ incubator. As a positive control, PBMCs were incubated with Concanavalin A (ConA).

### Analysis of IL12 production

The supernatants from the co-cultures of lymph cells and PBMCs were collected and used in an ELISA assay to measure IL12 levels. Maxisorp plates (NUNC) were coated overnight at 4 °C with a mouse monoclonal antibody against IL12 in carbonate-bicarbonate buffer with a pH of 9.6. After washing, the plates were blocked with PBS-10% BSA for 1 hour at 37 °C. Undiluted supernatants from the co-cultures were added to the plates and incubated for 1 hour at room temperature. After washing, an optimal dilution of biotin-conjugated mouse anti-IL12 antibody was added and incubated for 1 hour at room temperature. Following another round of washing, an optimal dilution of horse radish peroxidase (HRP)-conjugated streptavidin (KPL^®^) was added to the plates. The plates were incubated for 1 hour at room temperature and then washed. Ortho-phenylene-diamine (OPD)/H_2_O_2_ substrate solution was added, and the reaction was allowed to develop. The reaction was stopped using 1.25 M H_2_SO_4_, and the absorbance at 492 nm (A492) was measured using a microplate reader. A positive control supernatant, obtained from lymph cells incubated with PolyIC, was included in the assay. The cutoff value was determined as the mean of the values from blank wells plus two standard deviations.

### Detection of Infectious viral progeny

Lymph cells or BHK-21 cells (positive control) were incubated on ice for 40 minutes with infectious FMDV serotype A2001 or O1C or were mock-infected. After incubation, the cells were washed three times with acid PBS (pH = 5.5) to remove any non-internalized virus, followed by washing with fresh RPMI 1640 medium (Gibco) containing 10% FCS.

An aliquot of the incubated and washed lymph-derived cells was used to assess the presence of residual infectious virus. The cells were inoculated onto FMDV-susceptible BHK-21 cell monolayers and incubated for 48 h. No infectious virus was detected confirming the absence of detectable residual infectious virus under these experimental conditions. The cells were then cultured at 37 °C in a CO_2_ atmosphere for 48 hours. After the incubation period, the cell supernatant was collected and seeded, undiluted and in serial 1:10 dilutions, onto FMDV-susceptible BHK-21. The BHK-21 cells were further incubated for 48 hours and observed under a microscope for the presence of cytopathic effects. The viral titers were calculated using the Reed and Muench technique.

### Analysis of 3ABC expression

Lymph cells or BHK-21 cells (positive control) were incubated with infectious FMDV serotype A2001 or O1C, or were incubated with supernatant collected from uninfected BHK-21 cells (mock group), for 30 minutes on ice and then for 2 hours at 37 °C. After the incubation period, the cells were treated with Fix/perm buffer (BD) to fix and permeabilize them. Subsequently, the cells were washed with perm/wash buffer (BD) and intracellularly stained using a monoclonal antibody against the 3ABC non-structural FMDV protein. Following incubation and washing, an anti-mouse IgG-PE conjugated antibody was added (BD) to detect the bound primary antibody. Finally, anti-DEC205 FITC-conjugated and anti-CD11c APC-conjugated monoclonal antibodies were added to identify the ALDCs population (FSC^high^/DEC205+/CD11c+). The cells were fixed with 0.2% paraformaldehyde and acquired using a FACScalibur cytometer and analyzed using CellQuest software (BD). The analysis of 3ABC expression was performed on the FSC^high^/DEC205+/CD11c+ cell population.

### Assessment of cell viability

Lymph cells were incubated for 16 hours with infectious FMDV serotype A2001 or O1C or were incubated with supernatant collected from uninfected BHK-21 cells (mock group). After the incubation period, the cells were washed with ice-cold PBS, resuspended in binding buffer, and FITC-annexin V/propidium iodide solution was added (BD). After a 15-minute incubation, the cells were washed with binding buffer, and an APC-conjugated monoclonal antibody against DEC205 was added to identify ALDCs population. After 15 minutes incubation, the cells were washed and acquired using a FACScalibur cytometer and analyzed using CellQuest software (BD).

### Statistical analysis

To compare the data among groups, one-way analysis of variance (ANOVA) was performed, followed by Dunnett’s *post hoc* test to compare each treatment with the corresponding control. A p-value < 0.05 was considered statistically significant.

## Results

### Inactivated and infectious FMDV induce different profiles of co-stimulatory molecules in ALDCs

A schematic representation of the experimental procedure followed for calf vaccination, prescapular lymph node removal, and cannulation is shown in **Figure 1A**, as described in the Materials and Methods.

Lymph cells were incubated overnight (16hs) with infectious or inactivated Foot-and-Mouth Disease Virus O1C serotype (O1C). Both infectious and inactivated FMDV were used at an MOI of 5 to minimize the possibility that the viral dose would represent a limiting factor for the cellular response. For the inactivated virus, the MOI was calculated based on the infectious viral titer determined prior to inactivation. Then, the expression of CD40, CD86, and MHCII on ALDCs gate was subsequently analyzed. As shown in **Figure 1**, exposure to inactivated O1C resulted in a significant upregulation of CD40 and CD86 expression, whereas the infectious virus did not modify the expression levels of these co-stimulatory molecules. In contrast, infectious O1C induced a significant downregulation of MHC class II expression on ALDCs.

A similar analysis was performed using FMDV serotype A2001. As shown in **Figure 2**, inactivated A2001 increased the expression of CD40 and CD86 on ALDCs, while exposure to the infectious virus resulted in downregulation of CD40, CD86, and MHC class II. Poly I:C (unspecific ALDC stimulator) was included as a positive control, and as expected, it induced the upregulation of all evaluated molecules.

### Lymphocyte proliferation and IL-12 secretion are induced following incubation of lymph cells with inactivated FMDV

Lymph cells were incubated overnight with either infectious or inactivated FMDV, followed by co-culture with autologous CFSE-stained PBMCs to assess lymphocyte proliferation. As shown in **Figures 3A, B**, lymph cells exposed to inactivated FMDV from both O1C and A2001 serotypes, induced significant PBMC proliferation, whereas lymph cells incubated with infectious virus did not induce proliferation above mock levels. Concanavalin A, a T-lymphocyte mitogen, was included as a positive control, confirming the proliferative capacity of PBMCs.

**Figure 3:**
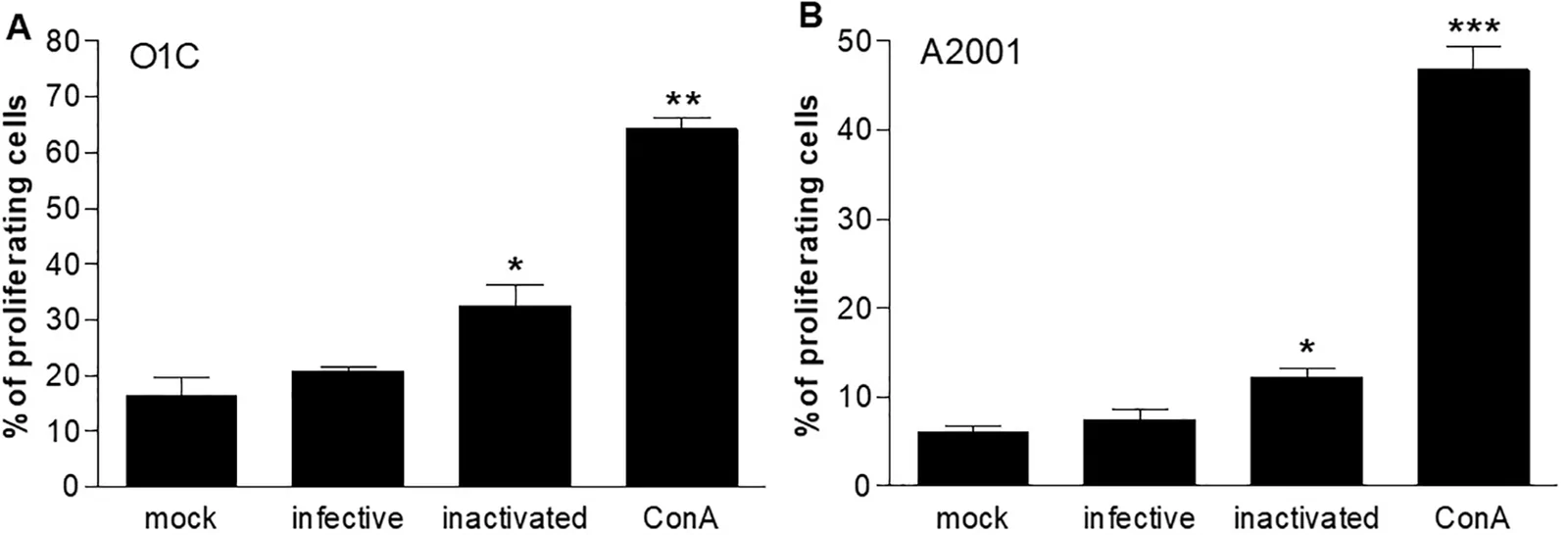
Percentage of proliferating cells after co-cultures with lymph cells incubated with infectious or inactivated virus from serotype **(A)** O1C or **(B)** A2001. “Mock”, “infectious”, or “inactivated” indicates the stimuli applied on lymph cells. Concanavalin A (ConA) mitogen was used as a positive control directly on PBMCs to assess the proliferative ability of PBMCs. * p < 0.05. **p < 0.01. ***p < 0.001.

To further characterize the response of the lymph cells, IL-12 levels were measured in co-culture supernatants by ELISA. As shown in **Figure 4**, significantly higher levels of IL-12 were detected only in supernatants from lymph cells incubated with inactivated FMDV, whereas infectious virus did not induce IL-12 secretion above mock controls. Poly I:C was included as a positive control for dendritic cell activation.

**Figure 4:**
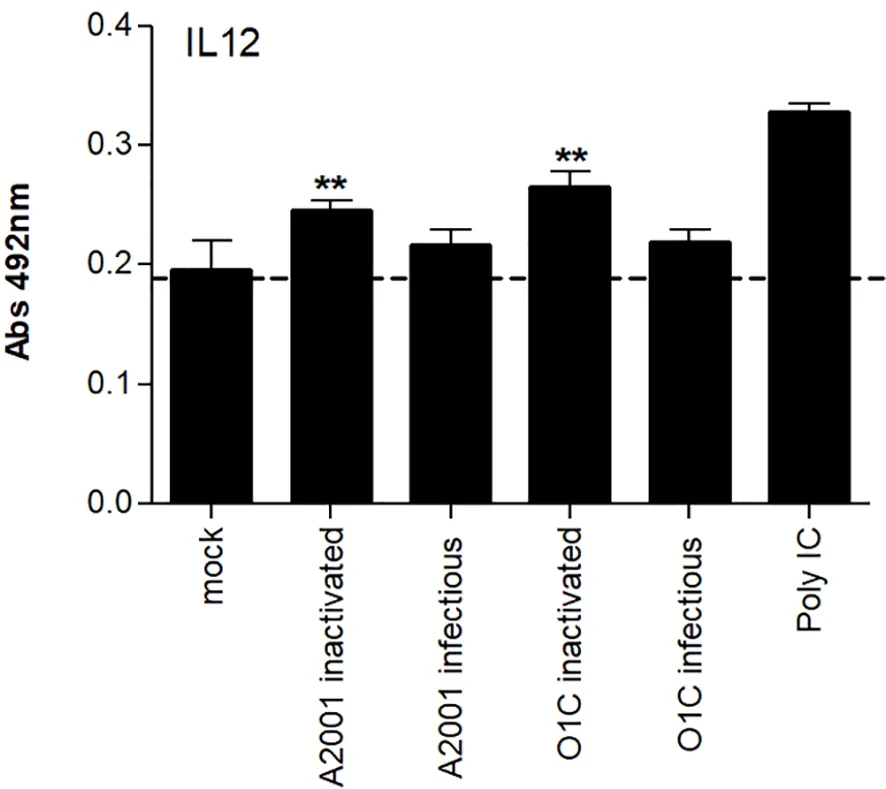
IL-12 secretion in co-culture supernatants was assessed by ELISA. Data are shown as mean Abs 492nm + SEM. The dotted line indicates the cut-off value. “Mock,” “infectious,” or “inactivated” indicates the stimuli applied to ALDCs. The “Mock” group consists of lymph cells stimulated only with complete culture media. Poly I:C was used as positive control for IL-12 production and was applied directly to lymph cells in the absence of PBMCs. **p < 0.01.

### Infectious FMDV fails to establish productive infection in lymph cells but A2001 induces limited viral protein expression in ALDCs

To determine whether FMDV could establish a productive infection in lymph cell cultures containing ALDCs, cells were incubated with infectious O1C or A2001 FMDV and subsequently subjected to an acidic PBS wash followed by several washes with PBS and culture medium, as described above. The washed lymph cells were then incubated in fresh culture medium, and the resulting culture supernatants were collected and inoculated onto FMDV-susceptible BHK-21 cell monolayers to assess the presence of infectious viral progeny. Viral titers in culture supernatants were assessed by infecting FMDV-susceptible BHK-21 cells. In parallel, BHK-21 cells were subjected to the same treatment as the lymph cells, including incubation with infectious FMDV, acidic PBS washing, and subsequent washes with PBS and culture medium. The supernatants from these BHK-21 cultures were then inoculated onto fresh FMDV-susceptible BHK-21 cell monolayers and served as a positive control to confirm that the washing and incubation procedures did not impair the production and detection of infectious viral progeny directly infected with FMDV and used as a positive control for productive infection.

As shown in **Figure 5A**, no detectable viral titers were observed in supernatants from lymph cell cultures exposed to infectious viruses compared to mock controls. In contrast, infection of BHK-21 cells using the same protocol resulted in high viral titers, confirming the efficiency of the infection and titration procedures.

**Figure 5:**
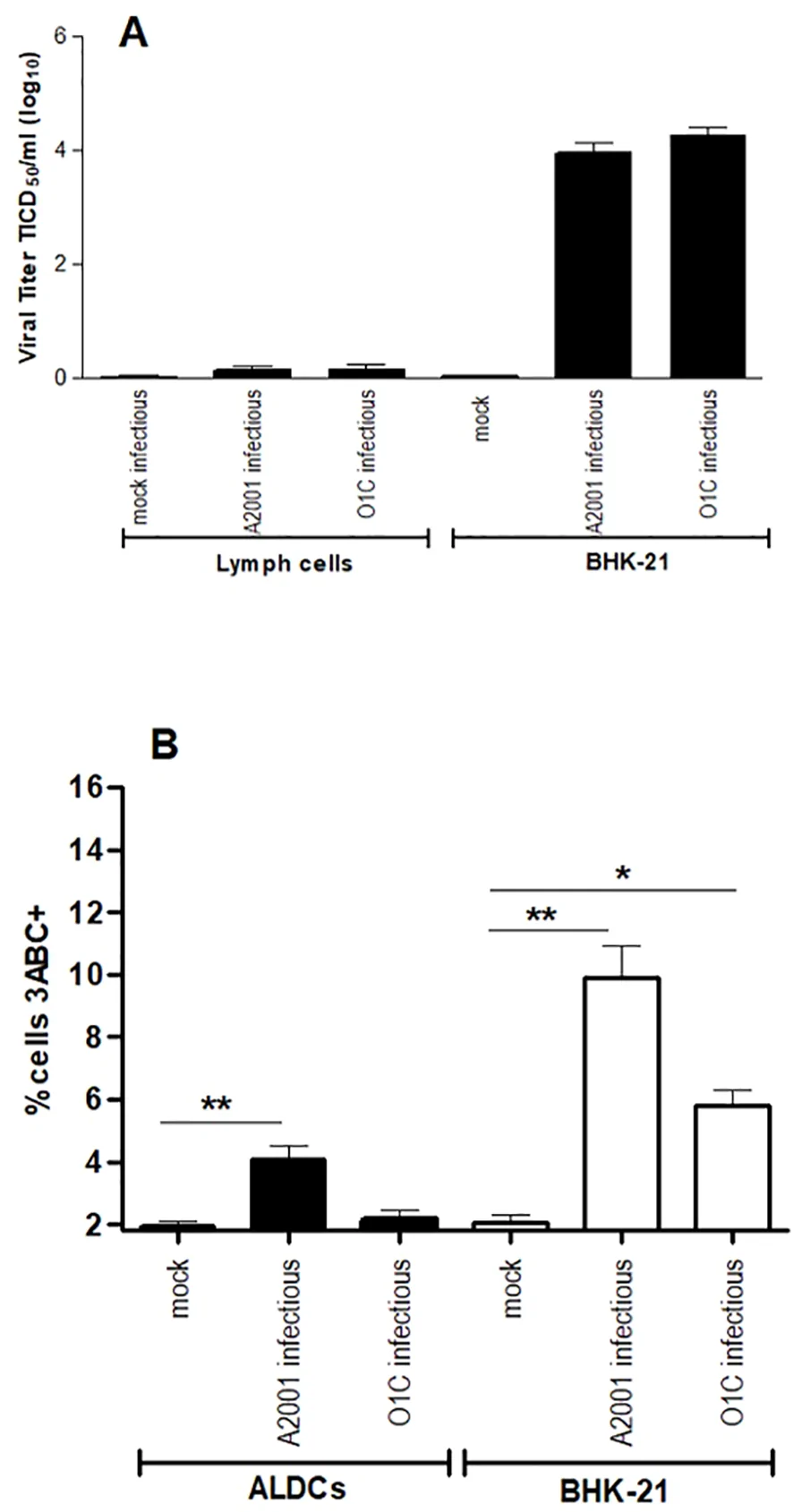
Viral replication in ALDCs cells. **(A)** Data are shown as mean viral titers ± SEM in the supernatants of lymph cells or BHK cells incubated with infectious virus. Mock control cells were subjected to the same experimental procedures as cells incubated with infectious virus, but without exposure to infectious FMDV. **(B)** Percentage of dendritic cells and BHK21 cells that were positive for 3ABC non-structural protein staining. *p < 0.05. **p < 0.01.

Since it has been reported that FMDV can undergo a single round of replication and/or protein expression in dendritic cells, we next assessed whether FMDV could express the nonstructural protein 3ABC in ALDCs. As shown in **Figure 5B**, 3ABC expression was significantly increased in ALDCs exposed to the infectious A2001 serotype, whereas no significant increase was detected in ALDCs incubated with the infectious O1C serotype. As a positive control, BHK-21 cells were infected and stained for 3ABC, demonstrating significant expression of this protein in FMDV-susceptible cells.

### Infectious FMDV serotype A2001 induces cell death in ALDCs

After assessing the pattern of co-stimulatory molecules expression and viral protein production in ALDCs incubated with the infectious A2001 serotype, we next evaluated whether infectious FMDV could affect ALDCs viability using annexin V/propidium iodide staining. Annexin V binds to phosphatidylserine, which is exposed on the outer leaflet of the plasma membrane during early apoptosis, while propidium iodide (PI) is a DNA-intercalating dye that enters cells with compromised membrane integrity, as occurs during late apoptosis or necrosis. Lymph cells were incubated with infectious O1C or A2001 FMDV and then stained with annexin V/PI. Early apoptotic ALDCs were defined as DEC205^+^/annexin V^+^ cells, whereas late apoptotic cells were defined as DEC205^+^/annexin V^+^/PI^+^ cells.

As shown in **Figure 6**, incubation with FMDV serotype A2001 resulted in a significant increase in apoptotic ALDCs, whereas incubation with the O1C serotype induces levels of cell death comparable to those observed in mock controls. Baseline levels of apoptosis detected in mock-treated cells are likely attributable to *in vitro* culture conditions.

**Figure 6:**
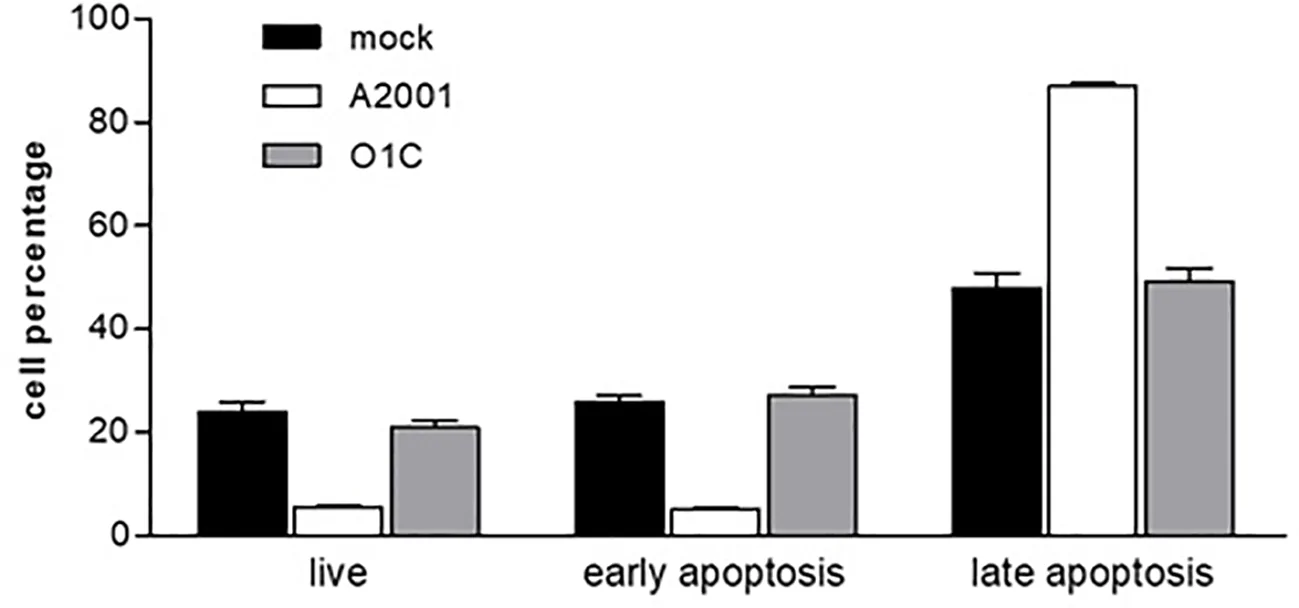
Viability of ALDCs following incubation with infectious FMDV: The percentage of ALDCs classified as viable (Annexin V^-^/PI^-^), in early apoptosis (Annexin V^+^/PI^-^), or in late apoptosis (Annexin V^+^/PI^+^) following incubation with infectious FMDV serotypes A2001 or O1C is shown. Mock-treated cells were incubated under identical conditions in the absence of virus.

## Discussion

Vaccination with inactivated formulations remains the primary strategy for controlling Foot-and-Mouth Disease. Conventional oil-inactivated FMD vaccines induce a strong but short-lived immune response, providing serotype-specific protection that correlates mainly with high titers of neutralizing antibodies. These antibody titers typically peak between 10 and 14 days post-vaccination and subsequently decline (28, 29). In contrast, natural FMDV infection induces a more rapid antibody response, with virus-specific antibodies detectable as early as 3–4 days post-infection, followed by a rise in IgM and later IgG titers (30) and the immune response elicited is generally more durable than that induced by vaccination (10, 12). These differences highlight that vaccination and natural infection engage the immune system through distinct mechanisms. Although these differences are well documented, the cellular mechanisms underlying these differences remain incompletely understood.

Because dendritic cells (DCs) are central regulators of T cell activation and adaptive immunity, their interaction with infectious versus inactivated virus may critically influence the trajectory of downstream immune responses. In the context of FMDV, previous studies have shown that the virus can modulate DC function in both murine models and natural hosts (31, 32).

Dendritic cells have been largely studied using populations differentiated *in vitro* from peripheral blood monocytes. Although this model is experimentally accessible and useful, *in vitro* differentiation may not fully reproduce the phenotype and functional properties of dendritic cells. In this context, we used bovine afferent lymph dendritic cells (ALDCs), a particularly relevant *ex vivo* model that reflects DC populations actively sampling peripheral tissues and migrating toward lymphoid organs, to examine how two different FMDV serotypes and two antigenic forms modulate DC activation and function. Although ALDCs provide a more physiologically relevant model, obtaining these cells represents a significant technical challenge. Successful cannulation and subsequent productive lymph collection depend on several factors, including the surgeon’s experience, the breed and anatomical characteristics of the animals, and appropriate post-surgical care and maintenance of the cannula. In our experience, the animals that presented the most favorable anatomical characteristics for cannulation were Holando-Argentino dairy cattle. During the training period with an experienced surgeon, successful cannulation was achieved in three out of four attempts. However, the cannulas remained functional for only a few days, and only one of the animals produced a substantial amount of lymph during this period. Subsequently, when the surgeon trained in this procedure performed the surgeries independently, the success rate decreased considerably. Nevertheless, in one successfully cannulated animal, improved fixation of the cannula together with appropriate post-surgical care allowed the cannula to remain functional for approximately one month, resulting in the collection of substantial amounts of lymph.

The objective of the present study was to compare the biological effects of the viral forms to which cattle are exposed during vaccination versus natural infection. Therefore, we employed two different virus propagation systems to obtain infectious and inactivated FMDV preparations to reproduce, as closely as possible, the viral forms encountered by cattle under vaccination and natural infection conditions. The inactivated FMDV corresponds to the vaccine strain propagated in BHK-21 cells and subsequently chemically inactivated, which is the formulation administered during vaccination. In contrast, the infectious FMDV was propagated in primary bovine thyroid cells to preserve the characteristics of a field virus and avoid cell-culture adaptations that may alter viral receptor usage and host-cell interactions. This experimental design was intended to reproduce biologically relevant conditions of natural infection and vaccination.

FMDV has evolved multiple immune evasion strategies that allow efficient replication and dissemination during early stages of infection. These include inhibition of host transcription and cap-dependent translation, interference with protein secretion, and cleavage of host signaling molecules (33).

The expression of MHCII and co-stimulatory molecules is essential for effective antigen presentation and T cell activation. MHCII-mediated peptide presentation enables antigen-specific recognition by CD4^+^ T cells, while CD86 and CD40 provide critical co-stimulatory signals required for T cell activation, expansion, and differentiation (34–36). In the present work, we evaluated the expression of CD40, CD86, and MHCII molecules at 16 h post-incubation, a time point selected based on previous studies reporting that changes in these markers can be observed during the 12–24 h period. (37, 38). In line with the known immune evasion mechanisms, incubation of bovine ALDCs with infectious FMDV serotypes O1C and A2001 resulted in downregulation of MHCII molecules or MHCII, CD86, and CD40, respectively, consistent with a functionally impaired dendritic cell phenotype. Similar downregulation of MHCII on DCs has previously been reported for other serotypes, including O1 Manisa and A24 Cruzeiro (39). On the other hand, cells stimulated with the inactivated virus upregulated CD40 and CD86 co-stimulatory molecules, while MHCII expression remained comparable to the mock group. It is noteworthy that exposure to inactivated FMDV did not result in an increase in MHCII expression, despite the upregulation of the co-stimulatory molecules CD40 and CD86. Although the mechanisms underlying the differential regulation of these molecules were not investigated in the present study, this observation is not necessarily unexpected, as CD40, CD86, and MHCII are not invariably regulated in a coordinated manner during dendritic cell activation. Previous studies have shown that the kinetics and magnitude of changes in co-stimulatory molecules and MHCII can vary depending on the dendritic cell population and the stimulus used (37, 38). Moreover, different temporal profiles of these markers cannot be excluded, and evaluation of additional time points may have revealed changes in ALDCs MHCII expression that were not apparent at the 16 h time point.

Despite the lack of increased MHCII expression lymph cells exposed to inactivated virus were associated with increased lymphocyte proliferation and IL-12 higher levels in co-culture assays. IL-12 is a key cytokine produced by DCs during antigen presentation and plays a central role in promoting CD4^+^ T cell expansion and differentiation (40, 41). However, since the co-cultures contained whole lymph-derived cells rather than purified ALDCs, the increased IL-12 levels detected in the supernatants cannot be attributed exclusively to ALDCs. Other cell populations present in the lymph-derived cell preparation or in the PBMCs may also have contributed directly or indirectly to the IL-12 levels detected in the co-culture supernatants.

The calves used for ALDC collection had been vaccinated with a commercial FMDV vaccine 5 months before lymph collection. This previous vaccination was necessary to allow a detectable antigen-specific proliferative response *in vitro*, since the low frequency of antigen-specific naïve T cells and the absence of the organized cellular interactions and lymphoid architecture required for efficient priming *in vivo*. Therefore, the lymphocyte proliferation observed in the co-culture experiments should be interpreted as a recall response rather than a primary T-cell response. In our system, lymph cells were exposed to virus and subsequently washed prior to co-culture, providing a setting in which antigen presentation could occur without the presence of free infectious virus. Nevertheless, because the proliferation assay was performed using lymph-derived cells rather than purified ALDCs, the observed response cannot be attributed exclusively to ALDC-mediated antigen presentation. Other antigen-presenting cells present in the lymph, such as monocytes or B cells, may also have contributed to antigen presentation following exposure to inactivated FMDV. Although increased lymphocyte proliferation was observed in co-culture assays, the specific lymphocyte subsets driving this response were not directly identified. Further characterization of these subsets, as well as the relative contribution of individual antigen-presenting cell populations, would provide additional information about the functional consequences of FMDV-mediated modulation of lymph-derived cells. Nevertheless, under the experimental conditions used here, exposure of lymph-derived cells to inactivated FMDV was associated with increased PBMC proliferation, whereas exposure to infectious FMDV did not produce a comparable response. Our proliferation results are in line with previous findings in cattle showing that the immune response to inactivated FMDV antigen strongly relies on T-cell collaboration (42), whereas the primary antibody response following FMDV infection can proceed independently of T-cell help (43). These findings are also in agreement with previous studies in a murine model, in which Ostrowski and colleagues demonstrated that FMDV can infect murine DCs *in vitro* and subsequently suppress thymus-dependent immune responses *in vivo* (15, 16).

Both apoptosis and infection by pathogens are known to trigger the downregulation of co-stimulatory molecules. To determine whether the observed downregulation was related to either of these two phenomena, further investigation was carried out. Some viruses, such as HIV, CSFV, and influenza, induce DC apoptosis as a mechanism to evade host immune surveillance, providing a window period for viral replication and propagation (44). In a murine model, our group demonstrated that FMDV induces DC death through the intrinsic apoptotic pathway, specifically by activating caspase-9 via ERK1/2 phosphorylation (45). We initially assessed whether FMDV replicates in dendritic cells, as viral replication, given the cytopathic nature of FMDV, could potentially lead to dendritic cell destruction. However, as shown in **Figure 6A**, no viral titers were detected in cultures of lymph cells, which comprise both ALDCs and lymphocytes. These results are consistent with previous findings (8, 46, 47).

Since it has been reported that FMDV can undergo an abortive replicating cycle in DCs, we examined the presence of the non-structural protein 3ABC in ALDCs. 3ABC is the precursor of 3A and antibodies against this polyprotein is a well-established marker of viral replication and infection *in vivo* (8, 48). Our results revealed a significant increase in the 3ABC protein in ALDCs incubated with the infectious A2001 serotype, while no 3ABC was detected following incubation with the infectious O1C serotype.

Although infectious FMDV did not result in productive viral replication in lymph cell cultures, the detection of the non-structural protein 3ABC in ALDCs, particularly following incubation with the infectious A2001 serotype, suggests that FMDV can initiate viral protein expression without generating infectious progeny. The detection of 3ABC should nevertheless be interpreted with caution and does not, by itself, demonstrate the completion of the viral replication cycle. FMDV has a positive-sense RNA genome that can serve directly as a template for protein translation once available in the cytoplasm. Therefore, detection of a viral non-structural protein may occur without progression to efficient genome replication, synthesis of structural proteins, assembly, and release of infectious progeny. This interpretation is consistent with our observation that, despite the detection of intracellular 3ABC in A2001-exposed ALDCs, infectious progeny could not be recovered from lymph cells.

Previous studies have reported variable results regarding FMDV infection of lymphocytes and DCs. While Díaz-San Segundo and colleagues (12) reported that FMDV serotype C can infect T and B cells, contributing to the observed T-cell depletion, and Joshi and colleagues demonstrated FMDV replication in lymphocytes through RT-PCR analysis (49), our results, together with those of Bautista et al. (17), support a lack of productive infection. Differences among these studies may reflect variation in serotypes, viral adaptation, and cell types used. For example, Robinson et al. (8) showed that integrin-binding virus failed to infect moDCs, whereas cell culture-adapted virus could do so, underscoring the importance of viral phenotype.

Previous studies have also demonstrated that FMDV isolates can differ in their replication properties and pathogenicity. For example, despite being closely related, the A/Argentina/2000 (A/Arg/00) and A2001 isolates have been reported to differ in lesion severity in cattle, infectivity in mice, and viral growth in cell culture. Studies comparing these isolates have identified genetic and functional differences affecting viral replication, including differences in IRES structure and IRES–3′UTR activity. Since the IRES is responsible for the internal initiation of viral RNA translation, these findings indicate that strain-specific differences may affect viral protein expression and viral replication through mechanisms acting downstream of viral entry. Thus, although these studies do not directly explain the differences observed between A2001 and O1C in our ALDC model, they support the possibility that differences in intracellular events following viral entry may contribute to the distinct phenotypes observed in our study.

In the present work, we did not directly assess viral binding, internalization, viral RNA replication, or the expression of specific integrin receptors on ALDCs. Therefore, our data do not allow us to determine whether A2001 has a higher affinity for ALDC surface integrins, whether it utilizes alternative co-receptors compared with O1C, or whether ALDCs are effectively infected by FMDV. In addition, although previous reports have associated abortive infection with a lack of ALDC maturation, as reflected by reduced MHCII expression and the absence of co-stimulatory molecule upregulation, lymphocyte proliferation, and IL-12 secretion, the mechanisms underlying the distinct responses observed in our study remain to be determined.

Regarding O1C serotype, our data suggests that it cannot translate non-structural proteins in ALDCs or lymphocytes. Nonetheless, we cannot rule out the possibility of abortive replication occurring in ALDCs incubated with infectious O1C, as more sensitive technics such as RT-PCR for detecting the negative strand of viral RNA, were not employed in this study.

Assessment of cell death revealed additional differences of serotype-dependent modulation. Only the A2001 serotype induced significant ALDC apoptosis, as detected by annexin V/propidium iodide staining. Given the absence of productive replication and the modest levels of 3ABC expression, this cell death is unlikely to result from classical cytopathic effects. Instead, it may reflect the impact of viral non-structural proteins, such as 2B and 2C, which are known to disrupt cellular membranes, alter Ca²^+^ homeostasis, induce autophagy, and engage apoptotic pathways (48). These findings are consistent with previous reports demonstrating FMDV-induced DC apoptosis in murine models (45) and with our observation that only the serotype capable of expressing non-structural proteins in ALDCs triggered cell death.

Taken together, our results indicate that infectious FMDV serotypes A2001 and O1C interfere with ALDCs function through distinct mechanisms. A2001 induces ALDCs death, whereas O1C may impair antigen presentation, as reflected by reduced MHCII expression and the lack of upregulation of co-stimulatory molecules. These findings are consistent with previous studies in mice and cattle, which show that FMDV O1C renders DCs poor stimulators of T cell responses while allowing the rapid generation of neutralizing antibodies through T-independent mechanisms (15, 42, 43).

Other RNA viruses also show distinct responses to infectious and inactivated forms, although the direction of the effect varies depending on the virus. In many cases, such as with Influenza A virus or replicating RNA vectors, exposure to the replicative form has been shown to induce stronger DC activation than the inactivated forms, in several studies (50, 51). However, when considering viruses more closely related to FMDV, such as Vesicular Stomatitis Virus (VSV), the pattern aligns with our observations: DCs fail to activate properly upon exposure to the infectious virus (52, 53). These findings support the idea that virus-specific factors, including replication strategies and immune evasion mechanisms, shape the nature of DC responses. Our findings clearly show that inactivated and infectious viruses exert markedly different effects on DCs. Nevertheless, further investigation is required to better understand FMDV-host interactions and to elucidate why infectious virus elicits a stronger and more long-lasting immune response than vaccination with the inactivated virus, despite its ability to evade the host immune system during the early stages of infection. Importantly, our results highlight a strong serotype-dependent modulation of dendritic cell function, underscoring that generalizations across serotypes may be misleading. Therefore, caution should be exercised when extrapolating findings from one serotype to another. Future studies including additional FMDV serotypes could help determine whether the observed differences are serotype-specific or represent a broader trend.

It is worth to note that although the use of cannulated ALDCs provides a physiologically relevant ex vivo model, as with other ex vivo experimental systems, the static culture conditions cannot fully reproduce the complex systemic, cellular, and tissue-level environment present during infection or vaccination in a living animal. The findings obtained in this ex vivo model should not be directly extrapolated to the *in vivo* context. Also, the use of cells from a limited number of donors does not allow us to assess the potential influence of individual biological variation or differences in baseline immune status on the observed responses. Validation using ex vivo cells from a larger and more diverse cohort of animals would be valuable in future studies.

In the present study, ALDCs were identified based on their FSC^high^/DEC205+/CD11c+ phenotype. It has been reported that these cells comprise heterogeneous subpopulations including four subsets with distinct phenotypic characteristics (defined mainly by the presence or absence of CD172a and CD1b) that differ in their capacities for antigen uptake and cytokine expression. (54, 55 and 24). Although a detailed characterization of ALDC heterogeneity and the functional contribution of individual subsets was beyond the scope of the present study, identifying the specific ALDC populations involved in the responses observed here could provide valuable additional information. Such analyses could help determine whether the effects of FMDV exposure are broadly shared among ALDC subsets or preferentially associated with specific populations. Further studies addressing bovine ALDC heterogeneity may therefore provide a better understanding of how distinct subsets respond to FMDV.

Overall, the findings of the present study provide new insights into the differential effects of infectious and inactivated FMDV on bovine ALDCs and advance our understanding of the mechanisms through which FMDV modulates host immune responses. Further investigation of these mechanisms will contribute to defining their relevance to FMDV pathogenesis and immunity and may ultimately support the development of improved and rationally designed strategies for FMD control.

## Data Availability

The data supporting the findings are presented in the article. Further inquiries regarding the data may be directed to the corresponding author.
